# Fabrication of Refractive Index Tunable Coating with Moisture-Resistant Function for High-Power Laser Systems Based on Homogeneous Embedding of Surface-Modified Nanoparticles

**DOI:** 10.3390/molecules23051105

**Published:** 2018-05-07

**Authors:** Wei Yang, Xiangyang Lei, Haohao Hui, Qinghua Zhang, Xueran Deng

**Affiliations:** Research Center of Laser Fusion, CAEP, Mianyang 621900, China; hch890@163.com (W.Y.); leixiangyang2@163.com (X.L.); dream2001hui@163.com (H.H.); zhangqh502@sina.com (Q.Z.)

**Keywords:** moisture-resistant, refractive index tunable, high-power laser system

## Abstract

Moisture-resistant silicone coatings were prepared on the surface of potassium dihydrogen phosphate (KDP) crystal by means of spin-coating, in which hydrophobic-modified SiO_2_ nanoparticles were embedded in a certain proportion. The refractive index of such coating can be tuned arbitrarily in the range of 1.21–1.44, which endows the KDP optical component with excellent transmission capability as well as the moisture proof effect. A dual-layer anti-reflective coating system was obtained by covering this silicone coating with a porous SiO_2_ coating which is specially treated to enhance the moisture resistance. Transmittance of such a dual-layer coating system could reach 99.60% and 99.62% at 1064 nm and 532 nm, respectively, by precisely matching the refractive index of both layers. Furthermore, the long-term stability of this coating system has been verified at high humidity ambient of 80% RH for 27 weeks.

## 1. Introduction

As the frequency converting optical component for the high-power laser system of the inertial confinement fusion (ICF) facility, water-soluble potassium dihydrogen phosphate (KDP) crystal needs a coating system to achieve both anti-reflective and moisture-resistant function, in order to ensure its high laser transmission efficiency and long-term stability. The high-power laser system requires KDP components to possess high transmittance at 1064 nm and 532 nm to double/triple the frequency of original incident laser, which could realize high output power to implement final ignition of the ICF facility. Therefore, a coating system with required refractive index (RI), thickness, and the moisture-resistant function should be acquired for each layer.

Silica coating prepared using the sol-gel technique is a favorable candidate for the high-power laser system owing to its high laser resistant ability compared with coatings prepared by physical vapor deposition (PVD) [1,2,3]. The double-wavelength anti-reflective coating system for 1064 nm and 532 nm using the sol-gel process has been proposed by Thomas for the first time [4]. Subsequent studies implemented the coating preparation based on this theoretical design by fabricating the bottom layer with silicone coating (due to its dense structure and higher RI) using Si-O oligomer sol as a source [5], and the top layer with porous silica coating (owing to its porous structure and lower RI) using monodisperse colloidal silica nanoparticles (NPs) [6,7]. However, the RI of conventional synthesized silicone coating is higher than that of the design value because RI is an intrinsic characteristic of a material, which will result in transmittance reduction at desired wavelengths. Zhang et al. improved the method to prepare this double-wavelength anti-reflective coating system and transmittance; about 99.6% and 99.8% at 532 nm and 1064 nm were achieved, respectively [8]. The RI of top layer, however, is as low as 1.14 with a porosity about 73%. Generally, larger coating porosity means a larger possibility to absorb water vapor and contaminants in the environment [9,10], which also will lead to the transmittance reduction in long-term running of the component. Another RI modulation approach was proposed by Ye et al., in which the RI was tuned by mixing the base-catalyzed silica and acid-catalyzed silica sols in different proportions [11], but the precise control of pH is a potential challenge for this method because acid coating is harmful to the KDP substrate. Cai et al. reported a method to fill silica NPs in the silicone coating to modulate its RI, and the durability has been proven to be satisfactory [12]. However, the important moisture prevention of coating systems is not specifically discussed, which is very crucial to keep the long-term stability of KDP substrate. In summary, dense silicone coating with required RI is hard to obtain due to the intrinsic feature of raw materials, and silica NPs with moisture-resistant ability need extra treatment to function. An approach to modulate the RI of silicone coating precisely and enable both coatings with moisture-resistant ability is proposed in this study, based on the homogeneous embedding of the silicone coating and surface modification of the porous silica coating. A series of dual-layer double-wavelength broadband coating systems on KDP substrate were designed and a KDP component with excellent transmission properties and long-term stability was successfully fabricated.

## 2. Results and Discussion

### 2.1. Design of the Double-Wavelength Anti-Reflective Coatings

Design of this dual-layer coating system with double-wavelength anti-reflective function on KDP substrates was performed using the thin film design software (TFCalc^®^) and the optimized results are listed in Table 1. The RI was set to be variable from 1.10 to 1.44, and the starting thickness was set to be 100 nm for both layers. The optimal anti-reflective coating with a transmittance of near 100% at target wavelengths can be obtained while the RI and thickness was about 1.30 and 135 nm for the bottom layer, and around 1.14 and 154 nm for the top layer. However, the bottom layer with a low RI of 1.30 has a poor abrasive-resistance [13,14], while the top layer with such low RI of 1.14 was prone to absorb organic contaminants or water vapor from the environment [9,10], which will cause unexpected transmittance loss. To solve these problems, relatively high and compromised RI of 1.38 and 1.22 were adopted for the bottom and top layers, respectively. This pair of matched RIs can still achieve high transmittance around 99.7% at 532 nm and 1064 nm of the dual-layer coating system, while the abrasive-resistance for the bottom layer and anti-contamination for the top layer could be ensured.

### 2.2. Fabrication and Characterization of Top Layer for the Double-Wavelength Anti-Reflective Coating System

**Scheme 1:**
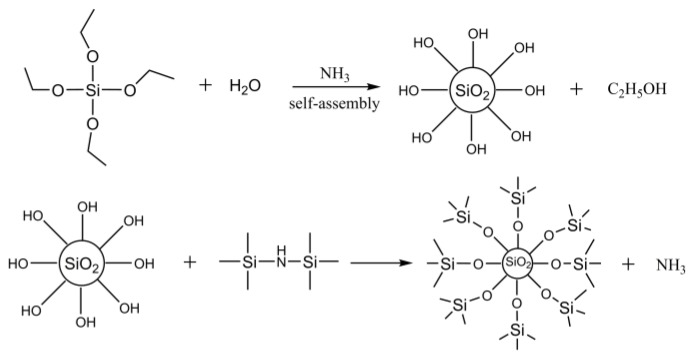
Synthetic routes of HMDS-modified silica oligomer sol.

The top layer is a porous silica coating consisting of HMDS-modified silica NPs with the porosity of about 50% were fabricated by a familiar hydrolysis approach of a silane [14,15]. The synthetic route of the HMDS-modified silica NPs is presented in Scheme 1. Hydrolysis and condensation of TEOS is performed under alkali condition using ammonia as catalyst to form Si-O oligomer. Colloidal silica NPs are produced by the self-assembly of oligomer during the aging process. HMDS is then introduced to realize the graft of hydroxyl group with a hydrophobic methyl group. Then, a porous silica top layer is prepared using this oligomer sol by means of spin-coating.

**Figure 1:**
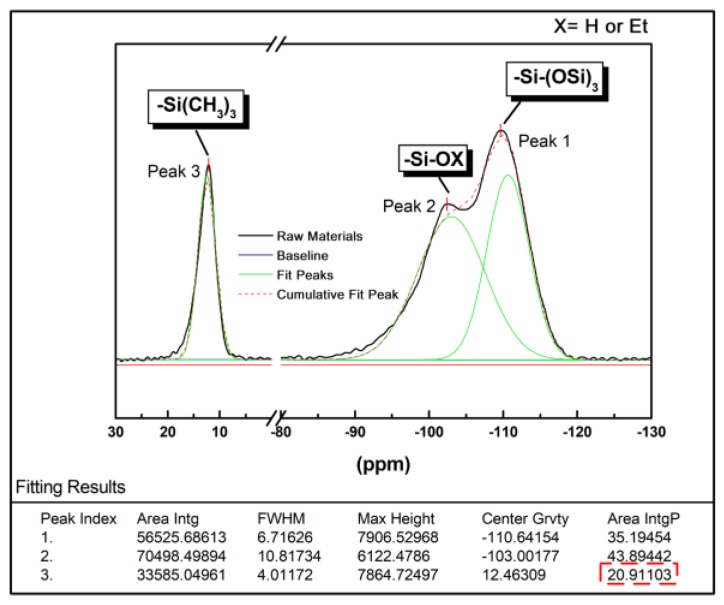
^29^Si MAS-NMR spectra of HMDS-modified silica NPs.

The modification of HMDS can be evaluated by calculating the grafting rate of trimethylsilyl (TMS, -Si(CH_3_)_3_) group via ^29^Si MAS-NMR as shown in Figure 1. Peak 1 represents the silicon atoms that have been totally condensed with only -OSi bond. Peak 2 implies the silicon atoms bonded with -OEt without hydrolysis or -OH without condensation. Peak 3 represents the silicon atoms bonded with -(CH_3_)_3_, indicating the effective HMDS modification with a ratio of about 20.9%. This grafting rate is reasonable according to the result of about 14–33% from the work of Suratwala et al. [16].

**Figure 2:**
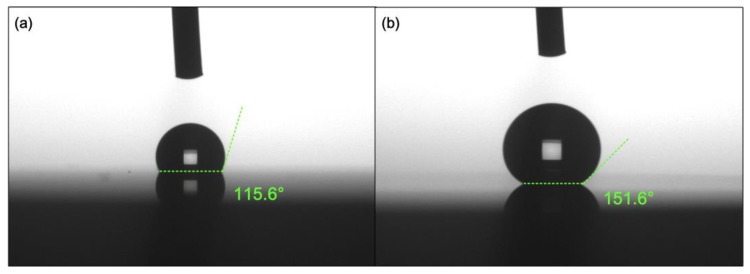
Water contact angle measurement results of solidified coatings from (**a**) As-synthesized colloidal silica sol; (**b**) HMDS-modified colloidal silica sol.

Moisture-resistance of this coating is surveyed via the water contact angle measurement as shown in Figure 2, in which solidified coatings prepared from as-synthesized colloidal silica sol and HMDS-modified colloidal silica sol are depicted and compared. Prominent improvement of the hydrophobicity of HMDS-modified porous silica coating is achieved by enlarging the water contact angle from 115.6° to 151.6°. This silica NP synthesis provides a favorable approach for further embedding modification of bottom silicone coating.

### 2.3. Fabrication and Characterization of Bottom Layer for the Double-Wavelength Anti-Reflective Coating System

**Figure 3:**
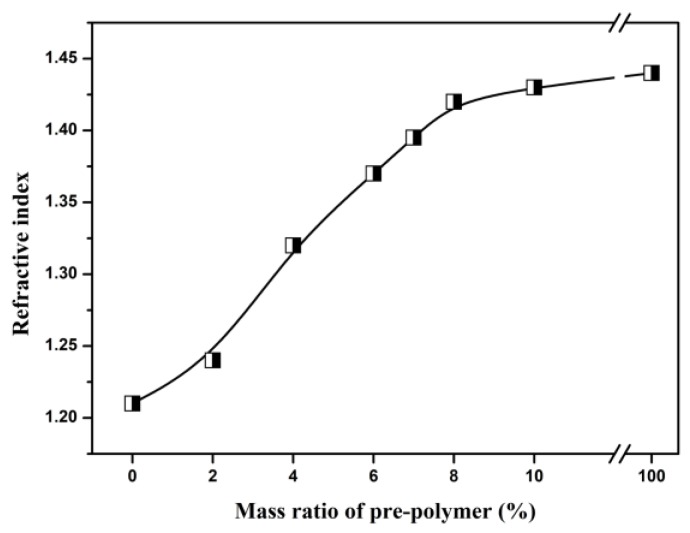
The relationship between the coating reflective index and mass ratio of pre-polymer.

The bottom layer in this study is a silica NP embedded silicone coating. As described above, the embedding of silica NPs is realized by blending the pre-polymer sol (33 wt %) and the HMDS-modified colloidal silica sol (3.3 wt %). Figure 3 demonstrates the relationship between coating RI and mass ratio of pre-polymer in the blended sol system, indicating that the RI of the bottom layer can be tuned from 1.21 to 1.44 with increasing of the concentration of pre-polymer. It also can be seen that the coating RI gradually tends to stabilize after the concentration of pre-polymer exceeds 10%. The blended sol with mass ratio of pre-polymer of about 6% (sol A) is qualified for the fabrication of the bottom layer in the double-wavelength anti-reflective coating system, as the solidified coating from this sol has the desired RI of about 1.38.

**Figure 4:**
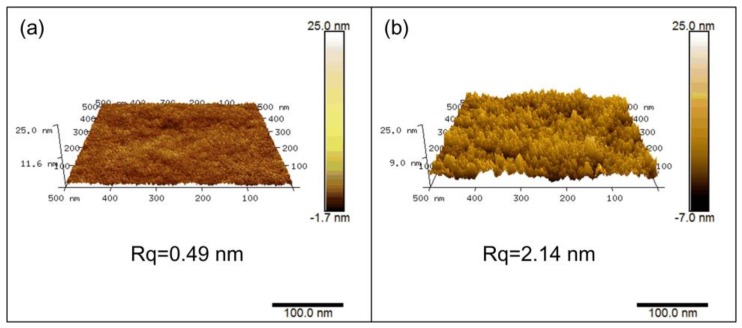
AFM images of solidified coatings from (**a**) As-synthesized pre-polymer sol; (**b**) Sol A.

AFM was utilized to characterize the microstructure of the embedded silicone coating as shown in Figure 4, in which obvious embedding structure of NPs can be observed. Figure 4a, and b demonstrate the morphology (in an area of 500 nm × 500 nm) of solidified coatings prepared from as-synthesized pre-polymer sol and sol A, respectively. The coating prepared from as-synthesized pre-polymer sol has a smooth surface with surface roughness Rq of 0.49 nm. The bump structure appears on the coating surface after the addition of silica NPs and the surface roughness Rq increases to 2.14 nm, indicating that the silica NPs were successfully embedded in the solidified silicone coatings.

**Figure 5:**
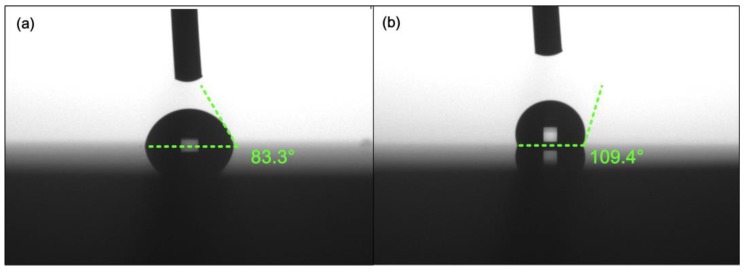
Water contact angle measurement of solidified coatings from (**a**) As-synthesized pre-polymer sol; (**b**) Sol A.

The embedding of HMDS-modified silica NPs into the bottom silicone coating is to modulate its RI to the designed value as well as to enhance its moisture-resistance capability. Water contact angle measurement results are given in Figure 5, in which solidified coatings from as-synthesized pre-polymer sol and sol A are compared. An increase from 83.3° to 109.4° is achieved after the embedding of HMDS-modified silica NPs, indicating successful enhancement of moisture-resistance of the bottom silicone coating.

### 2.4. Fabrication and Characterization of Dual-Layer Coating System with Double-Wavelength Anti-Reflective and Moisture-Resistant Function

**Figure 6:**
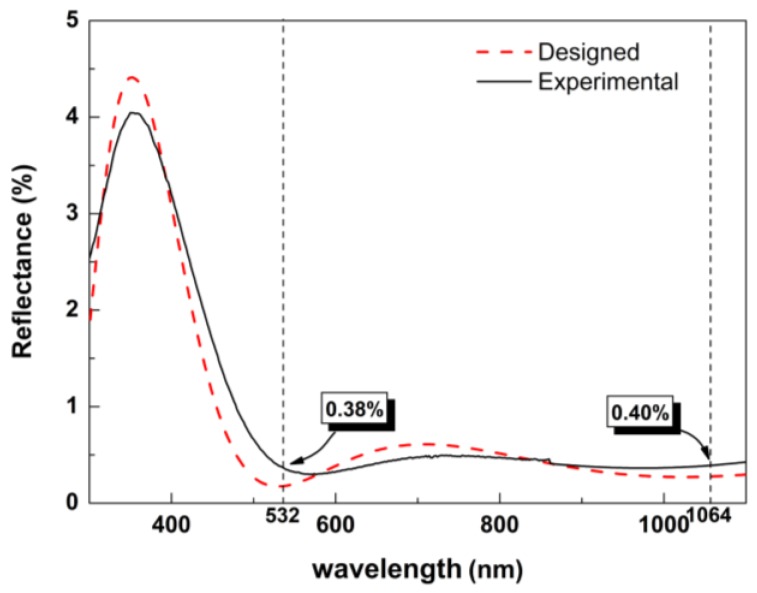
Reflectance spectra of fabricated dual-layer coating system and optimized theoretical design.

HMDS-modified silica NP embedded silicone coating (Coating A) was spin-coated and solidified on the KDP surface with an RI of 1.38 and a thickness of 130 nm as the bottom layer. Porous silica coating (Coating B) prepared using HMDS-modified NPs sol was then spin-coated over the Coating A with an RI of 1.22 and a thickness of 145 nm as the top layer. To evaluate the transmission property of the dual-layer coating system more precisely, the residual reflectance spectra is recorded via a spectrometer to exclude the effect of absorption from the KDP substrate. By introducing the coefficient of the standard reflective sample, reflectance spectra of this dual-layer coating system is depicted in Figure 6, in which the spectrum of a theoretically designed coating system is also given as a reference. Although the reflectance of this coating system is slightly higher than that of the optimized design at the desired wavelengths, the transmittance still reaches as high as 99.62% and 99.60% at 532 nm and 1064 nm, respectively, indicating successful modulation and exact matching of both layers’ RIs.

**Figure 7:**
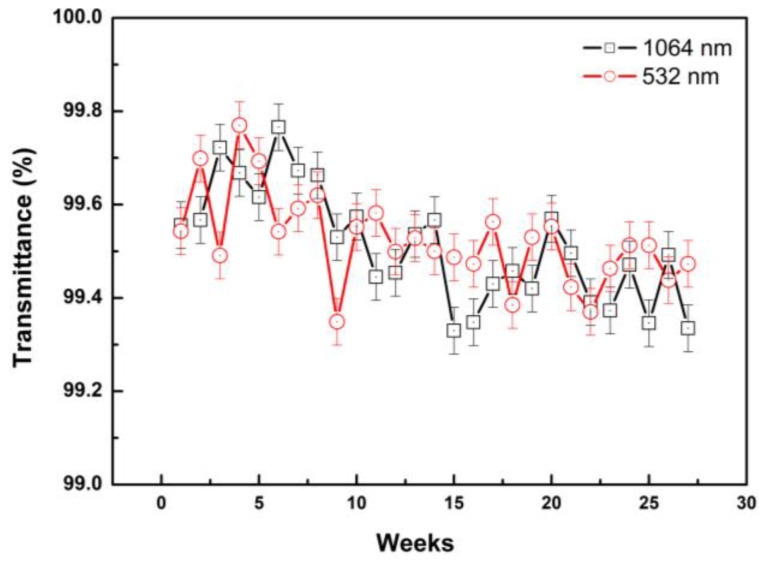
Transmittance variation of dual-layer coating system under 80% RH for 27 weeks.

Long-term stability of KDP component is verified by measuring its spectral property under an extreme condition of 80% RH every week. Figure 7 displays the transmittance variation of KDP component under 80% RH over 27 weeks. Only very few transmittance decrease appears in such a long period, implying successful enhancement of moisture-resistance by HMDS modification of silica NPs and embedding of the HMDS-modified silica NPs into the silicone coating.

## 3. Materials and Methods

### 3.1. Materials

Tetraethylorthosilicate (TEOS) was purchased from Sinopharm Chemical Reagent Co., Ltd. (Beijing, China). Methyltriethoxysilane (MTES), Hexamethyldisiloxane (HMDS), decane, and sec-butanol was purchased from Shanghai Aladdin Bio-Chem Technology Co., Ltd. (Shanghai, China). Anhydrous ethanol, heptane, and ammonia water were purchased from Chengdu Kelong Chemical Co., Ltd. (Chengdu, China). The water was deionized. The TEOS and MTES were distillation purified. Other chemicals were used without further purification.

### 3.2. Preparation of Silicone Pre-Polymer Sol

MTES (1783 g) that had been distillation purified, anhydrous ethanol (266.8 g), and water (586.8 g) were blended and stirred at 80 °C in a 5 L glass reactor with a distillation head, which could realize the distillation or reflux process in the same equipment. Pre-mixed water (198 g) and anhydrous ethanol (184 g) mixture were added when the solution started boiling. The resultant solution was distilled and refluxed for another 20 h, and then diluted with anhydrous ethanol to prepare pre-polymer sol with the concentration of 33 wt % after cooling to room temperature.

### 3.3. Preparation of Colloidal Silica Sol

The colloidal silica sol was prepared using TEOS (208 g) as matrix which was mixed with anhydrous ethanol (1610 g) and ammonium hydroxide aqueous solution (51.4 g, containing 30% ammonia) with stirring at 6 °C for 3 h. The solution was kept still in a sealed glass container for three to five days at room temperature to implement the aging process. It was then refluxed for 10 h to remove the extra ammonia. This colloidal suspension contained about 3.3% silica by weight in ethanol and was filtered through a 0.2-μm polyvinylidene fluoride (PVDF) membrane filter prior to use.

### 3.4. HMDS Modification of the Colloidal Silica NPs

The HMDS (180 g) was added into the refluxed colloidal silica sol (1818 g, 3.3 wt %), then stirring and aging were performed at room temperature for another 7 days. After that, decane (540 g) was added into the solution, and a sequential distillation at 120 °C was applied to this solution until all the ethanol and byproducts were removed.

### 3.5. Embedding of the Silicone Pre-Polymer with Modified Silica NPs

The silicone pre-polymer sol (33 wt %) and the HMDS-modified colloidal silica sol (3.3 wt %) were mixed in a certain proportion, and the RI of coatings prepared using this mixed sol can be were tuned according to the proportion.

### 3.6. Coating Preparation

The silicone pre-polymer and colloidal silica mixed sol was spin-coated on the surface of KDP substrates (50 mm × 50 mm × 10 mm) to fabricate the bottom layer under a velocity from 1000 to 1500 rpm at a rated time of 60 s to ensure that its thickness was around 130 nm at different mix proportion. After solidification of this bottom layer, the top layer was spin-coated using HMDS-modified colloidal silica sol under a velocity of about 300 rpm at a rated time of 80 s. The thickness of the top layer was around 150 nm after the solidification, in order to match the theoretical design of optimized conditions for the double-wavelength anti-reflective coating system.

### 3.7. Characterization

Ellipsometer (Sopra GES-5E, Courbevoie, France) was employed to measure the RI and thickness of coatings. The transmission spectra were recorded by a Perkin Elmer 950 UV-Vis spectrometer (Perkin Elmer, Waltham, MA, USA). Water contact angle was analyzed using Krüss DSA 100L (Krüss, Hamburg, Germany). The microstructural property of coatings was characterized using AFM (Bruker Dimension Icon, Karlsruhe, Germany). MAS NMR spectra were recorded in solid state using Varian Infinityplus 300WB (Varian, Palo Alto, CA, USA). The long-term stability verification was carried out in the BINDER KMF 115 cabinet (BINDER, Tuttlingen, Germany).

## 4. Conclusions

HMDS-modified porous silica coating with an RI of 1.22 and HMDS-modified silica NP embedded silicone coating with an RI of 1.38 have been prepared to fabricate a dual-layer coating system with double-wavelength anti-reflective and moisture-resistant function over KDP substrate. Successful and precise RI modulation of the silicone coating has been achieved by controlling the embedding ratio of HMDS-modified silica NPs. Transmittance values as high as 99.60% at 532 nm and 99.62% at 1064 nm of this coating system were realized and very few transmittance reduction occurred during a 27-week-long period under extremely high humidity of 80% RH.

## Figures and Tables

**Table 1:** Optimized design of a dual-layer coating system for double-wavelength anti-reflection.

Substrates	Bottom Layer			Top Layer		T_532 nm_ (%)	T_1064 nm_ (%)
	n	Thickness (nm)		n	Thickness (nm)		
KDP	1.26	136.7		1.12	162.5	99.92	99.91
	1.29	135.9		1.14	153.8	99.99	100.00
	1.30	135.0		1.14	153.6	100.00	100.00
	1.32	132.1		1.15	155.0	100.00	99.99
	1.34	130.6		1.16	152.7	99.98	99.96
	1.34	135.0		1.22	143.4	99.80	99.72
	1.36	129.1		1.17	150.6	99.95	99.90
	1.38	127.6		1.17	148.6	99.91	99.81
	1.38	130.5		1.22	143.4	99.78	99.67

n: All the refractive index discussed in this paper is at 632 nm.
